# Parametric and Semiparametric Approaches to Analyzing Device-Based Measures of Energy Expenditure in Zucker Diabetic Fatty Rats

**DOI:** 10.31083/j.fbl2802030

**Published:** 2023-02-20

**Authors:** Hyunkyoung Kim, Yuanyuan Luan, Roger S. Zoh, Guoyao Wu, Carmen D. Tekwe

**Affiliations:** 1 Department of Allergy and Clinical Immunology, Asan Medical Center, 05505 Seoul, Republic of Korea; 2 Department of Epidemiology and Biostatistics, Indiana University, School of Public Health, Bloomington, IN 47405, USA; 3 Department of Animal Science, Texas A&M University, College Station, TX 77843, USA

**Keywords:** energy expenditure, mixed effects models, spline regression, truncated splines, ZDF

## Abstract

**Background:**

Obesity results from a chronic imbalance between energy intake and energy expenditure. Total energy expenditure for all physiological functions combined can be measured approximately by calorimeters. These devices assess energy expenditure frequently (e.g., in 60-second epochs), resulting in massive complex data that are nonlinear functions of time. To reduce the prevalence of obesity, researchers often design targeted therapeutic interventions to increase daily energy expenditure.

**Methods:**

We analyzed previously collected data on the effects of oral interferon tau supplementation on energy expenditure, as assessed with indirect calorimeters, in an animal model for obesity and type 2 diabetes (Zucker diabetic fatty rats). In our statistical analyses, we compared parametric polynomial mixed effects models and more flexible semiparametric models involving spline regression.

**Results:**

We found no effect of interferon tau dose (0 vs. 4μg/kg body weight/day) on energy expenditure. The B-spline semiparametric model of untransformed energy expenditure with a quadratic term for time performed best in terms of the Akaike information criterion value.

**Conclusions:**

To analyze the effects of interventions on energy expenditure assessed with devices that collect data at frequent intervals, we recommend first summarizing the high dimensional data into epochs of 30 to 60 minutes to reduce noise. We also recommend flexible modeling approaches to account for the nonlinear patterns in such high dimensional functional data. We provide freely available R codes in GitHub.

## 1. Introduction

As sedentary lifestyles spread globally, obesity has become an increasing public health concern [1]. Sedentary lifestyles are growing rapidly with developing technology [2]. Obesity results from a chronic imbalance between food intake and energy expenditure, genetic predisposition, consumption of high fat diets, and inflammation [3]. Additionally, obesity contributes to adverse health outcomes such as insulin resistance, type 2 diabetes, obstructive sleep apnea, osteoarthritis, stroke, hypertension, and cancer [4]. As obesity becomes more prevalent, researchers seek to better understand the causal pathways leading to it. Energy expenditure is a key factor on these pathways and refers to the amount of energy used by the body for all physiological functions, such as movement, respiration, and digestion [5–7]. Energy expenditure has three components: resting metabolism, the thermic effect of feeding, and the thermic effect of physical activity [5,7]. Resting metabolism makes up 60% to 70% of an individual’s daily energy expenditure [5]. The thermic effect of feeding, including digestion, accounts for up to 10% of daily energy expenditure [5]. Finally, the thermic effect of physical activity comprises 20% to 30% of daily energy expenditure [5].

**Fig. 1.**
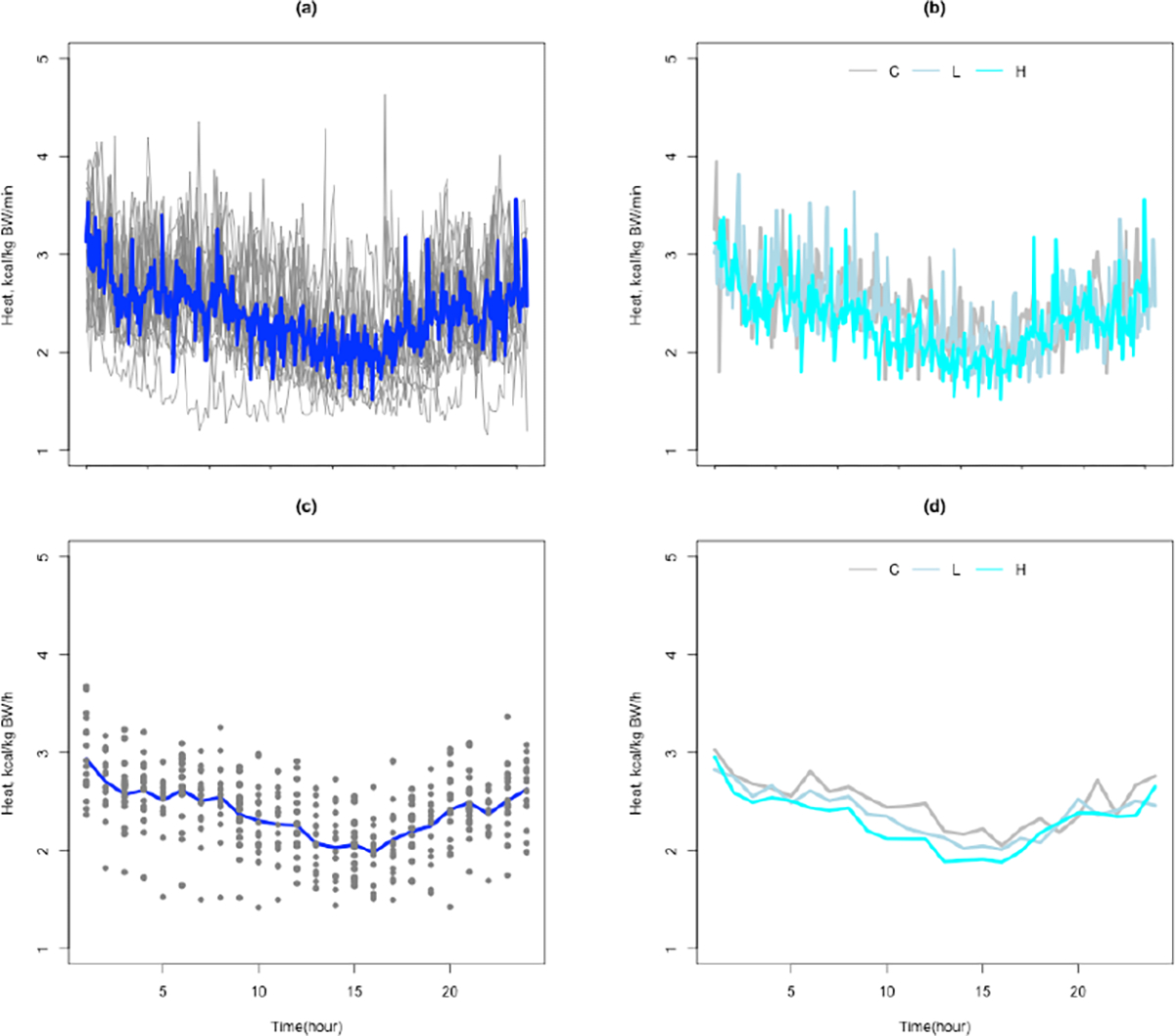
Plots of the heat production (kcal/kgBW/h) against time (in minutes and hours) over a 24 hour period for the 10-week-old ZDF rats. (a) shows the animal-specific trajectories of untransformed heat production in minutes. (b) shows the animal-specific trajectories of untransformed heat production in minutes by treatment group. (c) shows the animal-specific trajectories of untransformed heat production in hours. (d) shows the animal-specific trajectories of untransformed heat production in hours by treatment group. The blue lines in (a,c) are based on smoothing of the lines. In (b,d), C refers to the control group, L refers to the low dose group, and H refers to the high dose group.

Measuring energy expenditure accurately requires sensitive and sophisticated instruments. One commonly used instrument is the open circuit calorimeter, such as the computer-controlled Oxymax metabolic chamber for research animals (Columbus Instruments, Ohio, USA). This instrument measures energy expenditure in epochs of 60 seconds to five minutes during an observation period. The instrument calculates an animal’s energy expenditure from its volumetric carbon dioxide production (VCO_2_) and volumetric oxygen consumption (VO_2_). The device also records the animal’s total heat production (heat), and respiratory quotient (RQ). The resulting data are repeated measures that appear as curves or complex high dimensional non-linear functions of time (Fig. 1). Researchers often are confused about the most appropriate method for analyzing these data. A common approach is to compute a summary measure, such as the overall mean energy expenditure for the whole observation period or categorizing individuals on their intensity of activity [8–11]. These approaches are limited because they do not capture variation in energy expenditure or its pattern over time.

Because energy expenditure affects the development of obesity [12–14], some researchers have sought to manipulate energy expenditure and its physiological effects as a way to prevent or reduce obesity. Interferon tau, an anti-inflammatory cytokine, is one proposed intervention for achieving this aim [15, 16]. In a previous study, we evaluated the impact of interferon tau on obesity-related outcomes in Zucker diabetic fatty (ZDF) rats [11]. The ZDF animal model has deficiencies in its leptin receptors and therefore researchers often use it for obesity and type 2 diabetes studies. The objective of this study is to provide an introduction to more flexible approaches to assessing intervention effects on high dimensional data frequently collected in biomedical studies such as the device-based measures of energy expenditure.

## 2. Materials and Methods

We obtained 18 male 23-day-old ZDF rats from Charles River Laboratories and fed them a Purina 5008 diet throughout the study. The Purina 5008 diet consisted of 23.5% crude protein, 6.0% fat, 34.9% starch, 2.6% sucrose, 0.5% glucose plus fructose, 6.8% minerals, and 3.8% fiber, yielding 17,364 kJ gross energy/kg [11]. We kept the study animals in a temperature- and humidity-controlled facility on a 12-h light: 12-h dark cycle. The Texas A&M University Animal Use and Care Committee approved the study (#2010–251).

At 28 days of age, we randomly assigned the rats to receive drinking water (distilled and deionized H_2_O) with 0 (control), 4 (low dose), or 8*μ*g (high dose) of interferon tau/kg body weight per day (6 rats per condition). The rats had free access to food and drinking water during the 8-week study. To maintain assigned interferon tau dosages, we adjusted concentrations of interferon tau in the drinking water daily based on the volume of water the animals consumed. We changed their drinking water every other day. When the rats were 10 weeks old (week 6 of the interferon tau treatment), we placed each in an Oxymax chamber for 24 hours to assess energy expenditure. Approximately every five minutes, the instrument measured several indicators of energy expenditure: volumetric O_2_ consumption (VO_2_;L/h/kg body weight [BW]), volumetric CO_2_ production (VCO_2_; L/h/kgBW), respiratory quotient (RQ; CO_2_ production/O_2_ consumption) and heat production (kcal/h) (Heat). We focused our analyses on heat production. Our original report has further details on the experiment [11].

## 3. Models Considered

### 3.1 Linear Mixed Effects Models

Linear mixed effects models (LMEMs) can be used to analyze repeated measures data [17]. These models extend classical linear regression to correlated data. They provide powerful techniques for analyzing correlated data with complex variance structures, handling missing data, and incorporating nonlinear trends with log or higher order polynomial transformations. LMEMs take the following form:

(1) $$Y_{ij}=X_{ij}\beta +Z_{ij}b_{i}+\epsilon_{ij},$$

where $Y_{ij}$ is the $j^{th}$ response for the $i^{th}$ subject, β is a p×1 vector of fixed coefficients, $X_{ij}$ is a 1×p vector of fixed variables, $b_{i}$ is a q×1 vector for the random effects, and $Z_{ij}$ is a 1×q vector for the random variables. The random error terms $\epsilon_{ij}$ represent the random variation associated with the $Y_{ij}^{th}$ response. These models rely on the assumptions that $\epsilon_{ij}∼Normal\left(0,\sigma^{2}\right)$ and $b_{i}∼Normal(0,\;\Lambda )$, where Λ is the variance-covariance matrix for $b_{i}$. The mean response of $Y_{ij}$ is $X_{ij}\beta$, the fixed component of the model, while $Z_{ij}b_{i}$ is the random component of the model, representing individual variation from the overall sample mean and allowing description of individual-specific trajectories.

In assessing the impact of oral interferon tau supplementation on energy expenditure, we estimated 12 separate LMEMs resulting from all combinations of energy expenditure transformation (raw or log transformed), unit of time (minutes or hours), and time term (linear, quadratic, or cubic). We intended the log transformations to make the data approximately normal. We performed all analyses using the R Crans Software version 4.2.0 (R Core Team, Vienna, Austria) [18].

### 3.2 Semiparametric Mixed Effects Models

Penalized spline regression is a flexible semiparametric approach to estimating mean functions in mixed effects models [19]. Mean functions represented by splines can be expressed easily as the best linear unbiased predictors of the mixed effects model [20]. Semiparametric mixed effects models (SMEMs) are also specified as in Eqn. 1. However, the elements of the random components matrices differ from LMEMs. SMEMs include spline basis functions as random effects in addition to subject-specific random effects. Thus, SMEMs can be written as classical mixed effects models that include nonparametric terms for curve smoothing.

We used two kinds of semiparametric functions in our SMEMs: truncated power basis functions (TPBFs) and cubic B-spline functions.

#### 3.2.1 Truncated Power Basis Functions

Truncated power basis functions are simple semiparametric functions that approximate curves. We define a truncated power function at a given knot $\kappa_{k}$ as

$$\left\{\begin{array}{ll} {\left(x_{i}-\kappa_{k}\right)}^{p}=0 & x_{i}\leq \kappa_{k}\text{​}\\ {\left(x_{i}-\kappa_{k}\right)}^{p}>0 & x_{i}\geq \kappa_{k} \end{array}\right.$$

where p is the order of the polynomial function, and k=1,…,K represents the number of knots [21]. The functions are differentiable up to p-1 times [20–23]. In modeling mean functions, TPBFs approximate curves based on polynomial expansions. A mixed effects model based on truncated power basis is

(2) $$Y_{i}=X_{i}\beta +\beta_{0i}+\sum_{k=1}^{p}\;\alpha_{k}{\left(x_{i}-\kappa_{k}\right)}_{+}^{p}+\epsilon_{i}$$

where $Y_{i}$ is the $n_{i}\times 1$ vector of responses for the $i^{th}$ subject, $n_{i}$ represents the total number of responses per subject, $X_{i}\beta$ is the fixed part of the model, and $\beta_{0i}$ is the subject specific random intercept. The term ${\left(x_{i}-\kappa_{k}\right)}^{p}$ is a $p^{th}$ order truncated power basis of degree p, with $\kappa_{k}$ representing the $k^{th}$ knot [20]. Eqn. 2 is a polynomial piece-wise regression model with separate slopes, $\left(\alpha_{k}\right)$, fit to different partitions of the predictor variable. Thus, ${\left(x_{i}-\kappa_{k}\right)}_{+}$ is an indicator variable indicating the partition where ${\left(x_{i}-\kappa_{k}\right)}_{+}$ is positive. Knots are the points where adjacent partitions meet. For effective estimation, the TPBF approach requires an adequate number of knots or penalization [21].

Our cubic TPBF model of energy expenditure is

(3) $$\begin{array}{r} Y_{ij}=\beta_{0}+\beta_{1}\;*\;{time}_{i}+\beta_{2}\;*\;{time}_{i}^{2}+\beta_{3}\;*\;{time}_{i}^{3}\\ +\beta_{4}\;*\;{low}_{i}+\beta_{5}\;*\;{high}_{i}\\ +\sum_{i=1}^{k}\;\;\mu_{k}{\left({time}_{i}-\kappa_{k}\right)}_{+}^{3}+b_{0i}+\varepsilon_{ij} \end{array}$$

$\beta_{1},\;\beta_{2},\;\beta_{3}$ are the fixed coefficients for the linear, quadratic, and cubic terms for time, respectively, and $\beta_{4}$ and $\beta_{5}$ represent the low interferon tau and high interferon tau groups’ contrasts, respectively, with the control group. The ${\left({time}_{i}-\kappa_{k}\right)}_{+}^{3}$ term is the cubic spline basis. We treat the truncated cubic basis splines and the intercept, $b_{0i}$, as random and assume $\mu_{k}∼Normal\left(0,\sigma_{u}^{2}\right)$ and $b_{0i}∼Normal\left(0,\sigma_{b}^{2}\right)$. When $\sigma_{u}^{2}=0$, Eqn. 2 reduces to a mixed effects model. The random effects ${\left({time}_{i}-\kappa_{k}\right)}_{+}^{\left(p\right)}$, which we model as normal random curves with mean zero [23], are not present in the LMEM in Eqn. 1. The smoothness of the spline regression rises with increasing degree of the polynomial [23]. The smoothing parameter, $\lambda =\sqrt{\frac{\sigma_{u}^{2}}{\sigma_{\varepsilon }^{2}}}$, controls the smoothness of the curve, while the mean square error of the model grows with increasing λ [22,23]. Although easy to construct, models based on the TPBF can be numerically unstable due to correlations between the basis functions. When the range for $x_{i}$ in Eqn. 2 is wide, the basis functions increase rapidly as x rises. To resolve this issue, the range for $x_{i}$ may be re-scaled to [0, 1]. These disadvantages make the models prone to computational difficulties [21]. B-splines allow analysts to avoid these problems [21,24,25].

#### 3.2.2 B-spline Basis Functions

B-splines allow flexible approaches to analyzing data [21,25]. B-splines are piece-wise polynomial functions of order p connected at their inner knots [19,21,24,26]. While B-splines are equivalent to TPBFs on any given interval $\left[\kappa_{0},\kappa_{m}\right]$, they are more numerically stable [20,21,27]. B-splines are transformations of TPBFs [20,21]. To illustrate their equivalence, let $X_{T}$ and $X_{B}$ be design matrices for the TPBF and the B-spline basis functions of the same degree and same knot locations, respectively. Then $X_{B}=X_{T}L_{p}$ where $L_{p}$ is a square invertible matrix [20].

B-spline basis functions are nonzero over the interval $\left[k_{0},k_{m+1}\right]$. Next, let $\kappa =\left(\kappa_{0},\kappa_{1},\ldots ,\kappa_{m}\right)$ be a set of m+1 non-decreasing knots. The domain for B-splines is $\left[\kappa_{0},\kappa_{m}\right]$, with $k_{0}=0$ and $k_{m}=1$, typically representing the two boundary knots [24]. We define the $k^{th}$ *B*-spline basis function of degree p recursively as

$$\left\{\begin{array}{ll} B_{k,0}\left(\kappa \right)=1 & \;\kappa_{k}\leq \kappa \leq \kappa_{k+1}\text{​}\\ B_{k,0}\left(\kappa \right)=0 & \text{otherwise} \end{array}\right.$$

$$B_{k,p}(\kappa )=\frac{\kappa \;-\;\kappa_{k}}{\kappa_{k+p}\;-\;\kappa_{k}}B_{k,p-1}(\kappa )+\frac{\kappa_{k+p+1}\;-\;\kappa }{\kappa_{k+p+1}\;-\;\kappa_{k+1}}B_{k+1,p-1}(\kappa )$$

In our analyses, we specified the B-spline models as

(4) $$Y_{i}=X_{i}\beta +\beta_{0i}+\gamma_{i}B+\delta B+\epsilon_{i}$$

where $Y_{i}$ is the $n_{i}\times 1$ vector of responses for the $i^{th}$ subject, $X_{i}\beta$ and δB are the fixed effects, and $\beta_{0i}$ and $\gamma_{i}$ are the subject-specific random intercepts and random slopes for the B-spline basis functions, respectively.

### 3.3 Inference and Model Selection

One assumption of classical regression models is that covariates are independent. However, polynomial splines in regression models are not independent because they are piece-wise functions used to approximate curves. Therefore, the standard errors and confidence intervals for parameters in classical regression models are not applicable in models involving splines. For inference in spline regression models, nonparametric bootstrap methods can be used [28]. The nonparametric bootstrap involves resampling the data to estimate variances of model parameters without any distributional assumptions. To implement the nonparametric bootstrap, we first resampled the original data with replacement for each animal at different time points in the study. Next, we estimated model coefficients with the resampled data, and then repeated the resampling and estimation process *b* = 500 times. We computed the 95 th percent bootstrap confidence intervals using the percentile approach using $\left[Q_{\alpha /2},Q_{1-\alpha /2}\right]$, where α=0.05. The terms $Q_{\alpha /2}$ and $Q_{1-\alpha /2}$ represent the quantiles of the bootstrap distributions for the estimated coefficients.

We also calculated the corresponding p-values for the estimated coefficients under the null hypothesis of β=0 as $pvalue=2\;*\;min\left[Prob\left(\overset{ˆ}{\beta }<0|H_{0}\right),Prob\left(\overset{ˆ}{\beta }>0|H_{0}\right)\right]$ [28].

We selected models with the smallest Akaike information criteria (AIC) [29,30] values as the best fitting.

## 4. Results

Summarizing heat production by minute increased variability and random noise in the data relative to summarizing by hour (Fig. 1a–d). Heat production also varied nonlinearly over time. Other device based measures of energy expenditure showed similar patterns as heat production. Therefore, we focus our report on modeling heat production.

### 4.1 Linear Mixed Effects Models

**Fig. 2.**
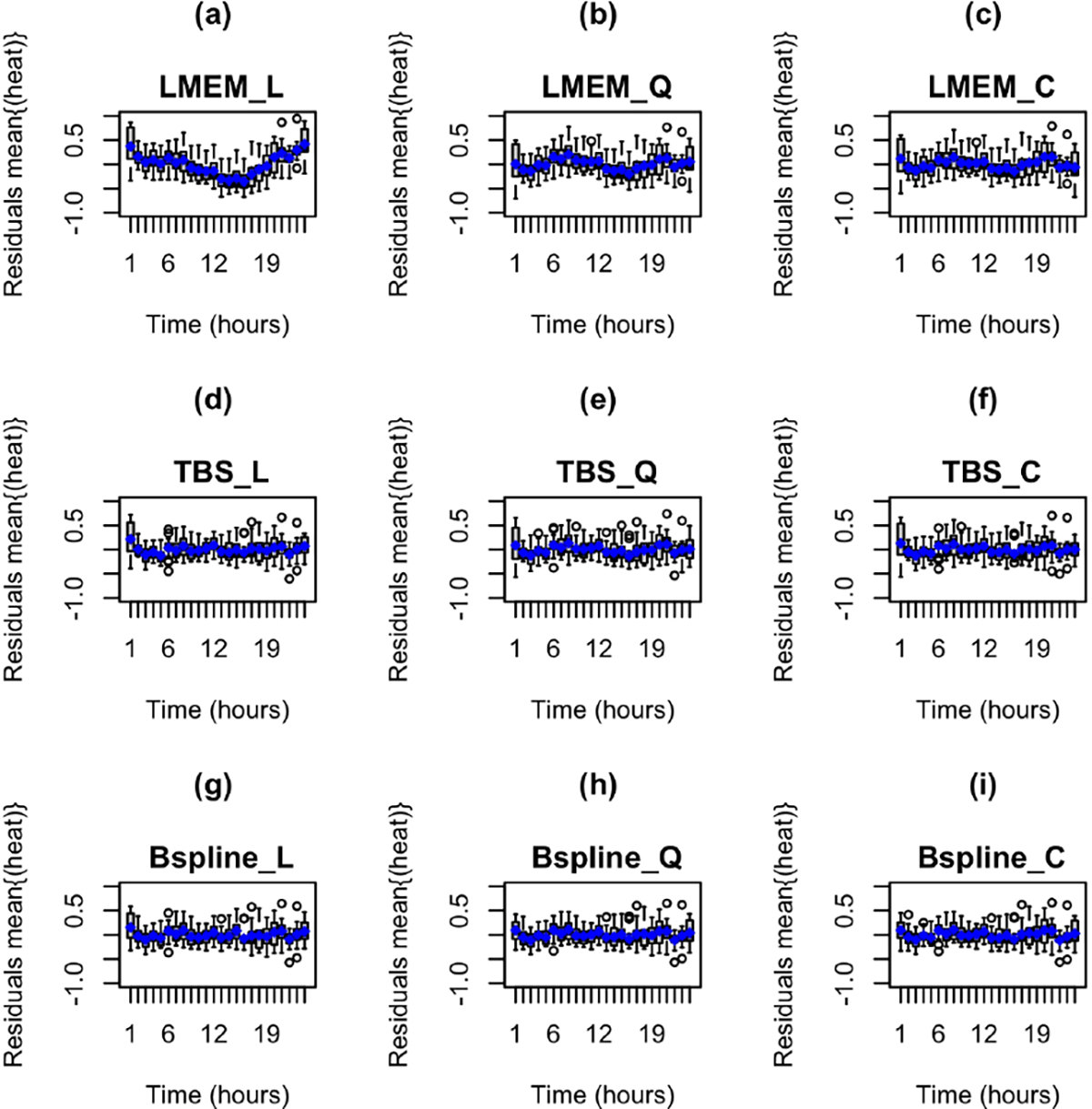
Boxplots of the residuals for heat production (kcal/kg BW/h) over time (hours) for 10-week-old ZDF rats. (a) LMEM with a linear term for time. (b) LMEM with a quadratic term. (c) LMEM with a cubic term for time. (d) TPBF SMEM with a linear smoothing spline. (e) TPBF SMEM with a quadratic smoothing spline. (f) TPBF SMEM with a cubic smoothing spline. (g) B-spline SMEM with a linear smoothing spline. (h) B spline SMEM with a quadratic smoothing spline. (i) B-spline SMEM with a cubic smoothing spline. The quadratic and cubic terms for time in the LMEMs fit the nonlinear trends in the data better than the LMEM with a linear term for time.

**Fig. 3.**
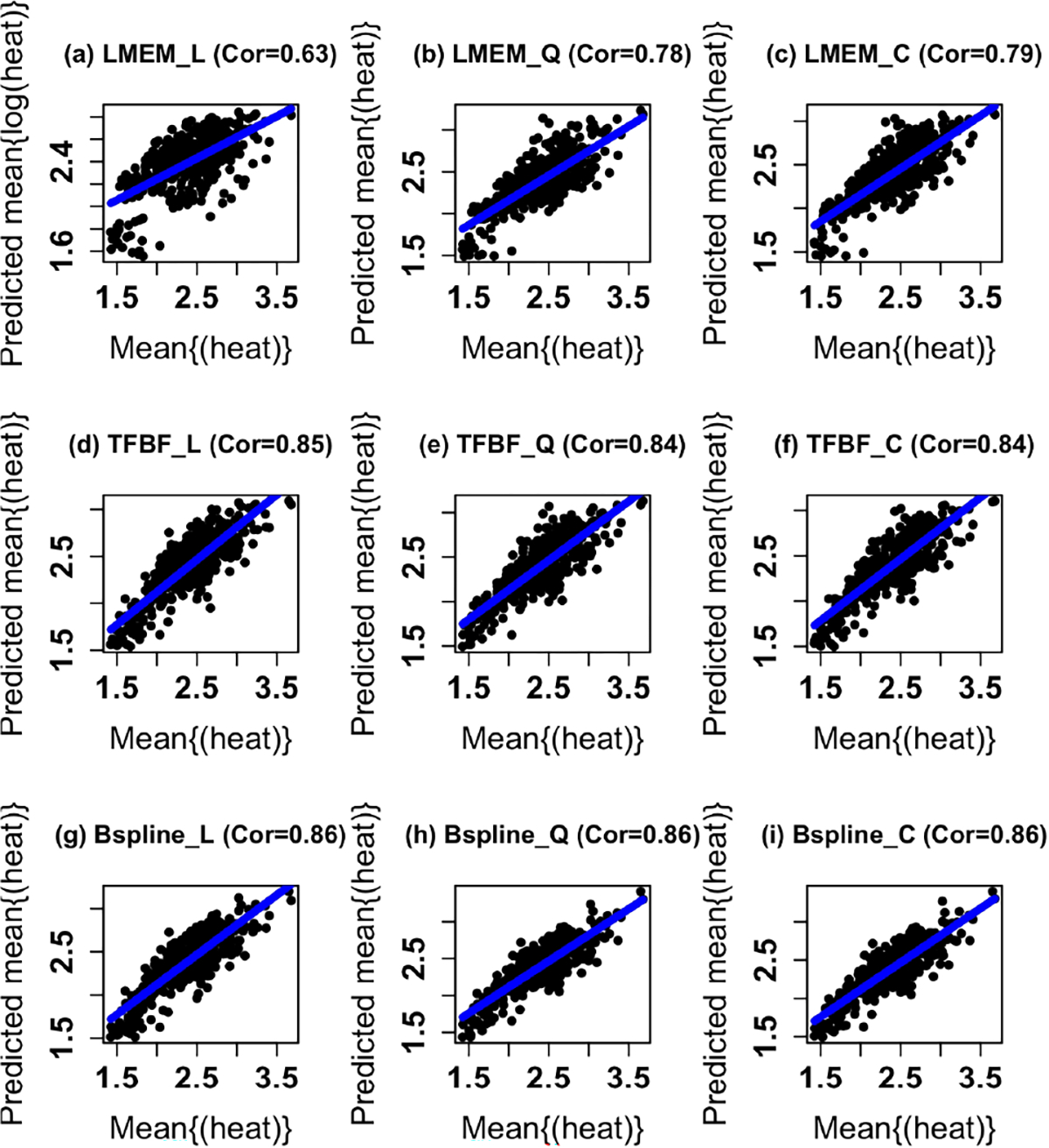
Predicted values of mean heat production ((kcal/kgBW/h) against observed mean heat production (kcal/kgBW/h) in 10-week-old ZDF rats. (a) LMEM with a linear term for time. (b) LMEM with a quadratic term. (c) LMEM with a cubic term for time. (d) TPBF SMEM with a linear smoothing spline. (e) TPBF SMEM with a quadratic smoothing spline. (f) TPBF SMEM with a cubic smoothing spline. (g) B-spline SMEM with a linear smoothing spline. (h) B spline SMEM with a quadratic smoothing spline. (i) B-spline SMEM with a cubic smoothing spline. The quadratic and cubic terms for time in the LMEMs fit the nonlinear trends in the data better than the LMEM with a linear term for time.

We estimated twelve separate LMEMs (Tables 1,2,3). The low and high dose groups did not differ significantly from the control group in heat production in any model. Models with a cubic term for time fit the data better than models with a quadratic term, which fit the data better than models with a linear term (see also Figs. 2a–c,3a–c). Also, models with log-transformed heat production fit much better than those with untransformed heat production. Furthermore, models of hourly mean heat production fit better than models of raw heat production at the scale of minutes, although the parameter estimates of paired models (differing only in time units) were very similar. Because coefficients for time terms and their standard errors were often close to the lower bound of zero, inference for these parameters may be inaccurate.

### 4.2 Semiparametric Mixed Effects Models

#### 4.2.1 Truncated Power Basis Functions

The TPBF models fit the data substantially better than the LMEMs (Table 4; Figs. 2d–f,3d–f). The linear spline TPBF model fit raw heat production at the scale of minutes best, while the quadratic spline model fit hourly mean heat production best. As in the LMEMs, there were no statistically significant treatment effects in any TPBF model. Also, TPBF models of hourly mean heat production fit better and had lower AIC values when compared to the AIC values for the analyses conducted at the minute-levels. For the cubic spline models, the higher order terms for time (quadratic and cubic) were not statistically significant. However, both the linear and quadratic terms for time were statistically significant quadratic spline models in the paired models that differed only in time scale.

#### 4.2.2 B-spline Basis Functions

The B-spline SMEMs (Table 5; Figs. 2g–i,3g–i) fit the data better than the TPBF models and LMEMs. As with all of the other models, there were no statistically significant treatment effects in the B-spline models. Also, B-spline models of hourly mean heat production fit better and had lower standard errors of coefficients than models of raw heat production at the time scale of minutes, although the patterns of coefficients for paired models were similar. The quadratic B-spline analyzed at the hourly level performed the best of all models we estimated for both mean hourly untransformed heat production and untransformed heat production at the time scale of minutes. Time was not statistically significant in the linear spline models for the analyses performed at the hourly and minute levels. However, the linear and quadratic terms for time were statistically significant in the quadratic spline model performed on a the hour level time scale. The quadratic and cubic terms for time were not statistically significant in the cubic spline models.

## 5. Discussion

Mixed effects models are useful for analyzing repeated measures data. However, with relatively noisy data such as device-based heat production data, variance parameters might not be well estimated. The semiparametric models, especially those with B-splines, approximated the nonlinear patterns in the untransformed heat production data better and thus had substantially higher predictive power than the parametric models. Another advantage of the semiparametric mixed effects modeling approach is that it does not require transforming the outcome variable (e.g., log transformation of heat production in our LMEMs) to make the data approximately normal and improve model fit. In analyzing energy expenditure data collected by devices, the first step is to evaluate plots of energy expenditure against time in minutes. If there appears to be a considerable amount of random noise in the plots, summarizing the data into longer time periods, such as hours, will reduce the random variation due to the frequency of data collection. If the data represent a high dimensional curve over time, rather than a linear function, we recommend semiparametric mixed effects models with smoothing splines for analysis.

In this manuscript, we demonstrated the use of semiparametric models to analyze noisy high dimensional data frequently collected by devices in epochs of 60-seconds over multiple days. A common approach to analyzing these data is to summarize the data into an overall summary such as overall heat production observed over a given week. In our previous analysis of these data [11], we summarized the data to the hourly level and used parametric linear mixed effects models to assess the effects of oral supplementation of interferon tau on device-based measures of energy expenditure. Our analysis included an interaction term between time and the treatment levels. The overall test for the interaction between treatments and time was not statistically significant at the 5% significance level. However, when separate analysis were conducted by each hour of observation to determine the treatment effects at each hour, we observed that the relationship between interferon tau treatment and measures of energy expenditure such as heat production depended on time and that the differences between the animals on the higher doses of interferon and the lower doses depended on time. A limitation of the current study is the sample size. The use of semiparametric methods in assessing treatment effects require larger sample sizes. Our findings have important implications for statistically analyzing data from experimental and clinical studies regarding effects of nutrition (e.g., dietary intakes of amino acids [31]) on improving metabolic profiles and health in animals and humans.

## 6. Conclusions

With the rise in complex data frequently collected from devices such as the Oxymas instrument, we recommend summarizing the data from units of time in minutes to hourly or half-hourly measures to reduce the noise associated with the frequency of data collection. The use of semiparametric regression methods provide more flexible modeling approaches to analyzing these data compared to parametric methods based on polynomial mixed effects models.

## Supplementary Material

EE_analysis_K_AIC_Github

EE_analysis_Github

## Figures and Tables

**Table 1.** Results for the LMEMs of heat production (kcal/kg BW/hr) with a linear time term.

Model	Outcome	Variable	$\overset{ˆ}{\beta }$	S.E.	*p*	AIC

LMEM (raw)	Heat	Intercept	2.589	0.058	<0.0001	4420.8
		L vs. C	−0.141	0.098	0.170	
		H vs. C	0.005	0.098	0.96	
		Time (mins)	−0.0003	0.00	<0.0001	

LMEM (log transformed)	*log(Heat)*	Intercept	0.933	0.027	<0.0001	−1921.06
		L vs. C	−0.06	0.05	0.21	
		H vs. C	0.011	0.05	0.81	
		Time (mins)	−0.0001	0.00	<0.0001	

LMEM *mean(Heat)/hr*	*mean(Heat)*	Intercept	2.584	0.063	<0.0001	328.575
		L vs. C	−0.141	0.097	0.169	
		H vs. C	0.015	0.097	0.877	
		Time (hr)	−0.015	0.002	<0.0001	

LMEM *mean{log(Heat)}/hr*	*mean{log(Heat)}*	Intercept	0.935	0.029	<0.0001	−398.918
		L vs. C	−0.061	0.045	0.201	
		H vs. C	0.015	0.045	0.749	
		Time (hr)	−0.006	0.001	<0.0001	

*p* is the parametric *p*-value obtained based on Z-score, which is given by $Z=\frac{\overset{ˆ}{\beta }}{S.E.}$.

C, control; L, Low dose; H, High dose; hr, hour; min, minutes

**Table 2.** Results for the LMEMs of heat production (kcal/kg BW/hr) with a quadratic time term.

Model	Outcome	Variable	$\overset{ˆ}{\beta }$	S.E.	*p*	AIC

LMEM (raw)	Heat	Intercept	2.992	0.06	<0.0001	3819.95
		L vs. C	−0.14	0.098	0.17	
		H vs. C	0.012	0.098	0.91	
		Time (mins)	−0.0002	0.0001	<0.0001	
		Time^2^	−0.000	0.000	<0.0001	

LMEM (log transformed)	*log(Heat)*	Intercept	1.110	0.028	<0.0001	−2554.46
		L vs. C	−0.06	0.05	0.21	
		H vs. C	−0.014	0.05	0.76	
		Time (mins)	−0.0001	0.000	<0.0001	
		Time^2^	0.000	0.000	<0.0001	

LMEM *mean(Heat)/hr*	*mean(Heat)*	Intercept	3.052	0.069	<0.0001	174.539
		L vs. C	−0.141	0.097	0.17	
		H vs. C	0.016	0.097	0.87	
		Time (hr)	−0.0124	0.008	<0.0001	
		Time^2^	0.004	0.0003	<0.0001	

LMEM *mean{log(Heat)}/hr*	*mean{log(Heat)}*	Intercept	1.133	0.031	<0.0001	−548.795
		L vs. C	−0.061	0.045	0.20	
		H vs. C	0.015	0.045	0.75	
		Time (hr)	−0.052	0.003	<0.0001	
		Time^2^	0.002	0.0001	<0.0001	

*p* is the parametric *p*-value obtained based on Z-score, which is given by $Z=\frac{\overset{ˆ}{\beta }}{S.E.}$.

Time^2^ is squared time (mins).

C, control; L, Low dose; H, High dose; hr, hour; min, minutes

**Table 3.** Results for the LMEMs of heat production (kcal/kg BW/hr) with a cubic time term.

Model	Outcome	Variable	$\overset{ˆ}{\beta }$	S.E.	*p*	AIC

LMEM (raw)	Heat	Intercept	2.87	0.063	<0.0001	3814.23
		L vs. C	−0.14	0.098	0.173	
		H vs. C	0.014	0.098	0.889	
		Time (mins)	−0.0001	0.0003	<0.0001	
		Time^2^	−0.000	0.000	<0.0001	
		Time^3^	−0.000	0.000	<0.0001	

LMEM (log transformed)	*log(Heat)*	Intercept	1.036	0.029	<0.0001	−2583.84
		L vs. C	−0.06	0.05	0.21	
		H vs. C	−0.015	0.05	0.74	
		Time (mins)	−0.0003	0.000	<0.0001	
		Time^2^	0.000	0.000	<0.0001	
		Time^3^	−0.000	0.000	<0.0001	

LMEM *mean (Heat)/hr*	*mean(Heat)*	Intercept	2.872	0.08	<0.0001	176.975
		L vs. C	−0.141	0.097	0.17	
		H vs. C	0.016	0.097	0.87	
		Time (hr)	−0.045	0.02	<0.0001	
		Time^2^	−0.003	0.0002	<0.0001	
		Time^3^	−0.0002	0.000	<0.0001	

LMEM *mean{log(Heat)}/hr*	*mean{log(Heat)}*	Intercept	1.045	0.036	<0.0001	−550.11
		L vs. C	−0.061	0.045	0.202	
		H vs. C	0.015	0.045	0.749	
		Time (hr)	−0.014	0.008	0.105	
		Time^2^	−0.002	0.0001	0.013	
		Time^3^	0.0001	0.000	<0.0001	

*p* is the parametric *p*-value obtained based on Z-score, which is given by $Z=\frac{\overset{ˆ}{\beta }}{S.E.}$.

Time^2^ is squared time (mins).

Time^3^ is cubic time (mins).

C, control; L, Low dose; H, High dose; hr, hour; min, minutes

**Table 4.** Results for TPBF models of heat production (kcal/kg BW/hr) with linear, quadratic, and cubic splines.

Model	Outcome	Variable	$\overset{ˆ}{\beta }$	p*	CI*	AIC

TPBF (linear spline)	Heat	Intercept	2.883	0.552	(−0.298, 3.247)	3447.53
		L vs. C	−0.108	0.508	(−0.352, 0.256)	
		H vs. C	0.088	0.588	(−0.255, 0.317)	
		Time (mins)	−0.002	0.648	(−0.004, 3.189)	

	*mean(Heat)*	Intercept	2.706	<0.0001	(2.576, 3.371)	179.369
		L vs. C	−0.106	0.300	(−0.387, 0.148)	
		H vs. C	0.045	0.944	(−0.248, 0.318)	
		Time (hr)	−0.017	<0.0001	(−0.004, −0.001)	

TPBF (quadratric spline)	Heat	Intercept	2.918	0.560	(−0.346, 3.233)	3505.01
		L vs. C	−0.051	0.456	(−0.406, 0.257)	
		H vs. C	−0.010	0.608	(−0.237, 0.314)	
		Time (mins)	−0.002	0.648	(−0.003, 0.000)^1^	
		Time^2^	0.000	<0.0001	(0.000, 3.18)	

	*mean(Heat)*	Intercept	2.981	<0.0001	(2.404, 3.274)	176.249
		L vs. C	−0.106	0.344	(−0.393, 0.253)	
		H vs. C	0.079	0.896	(−0.263, 0.358)	
		Time (hr)	−0.087	<0.0001	(−0.003, −0.001)	
		Time^2^	0.003	0.004	(0.000, 0.000)^1^	

TPBF (cubic spline)	Heat	Intercept	1.908	0.520	(−0.322, 3.277)	3601.67
		L vs. C	−0.250	0.512	(−0.393, 0.274)	
		H vs. C	−0.030	0.600	(−0.242, 0.35)	
		Time (mins)	−0.003	0.584	(−0.005, 0.000)^1^	
		Time^2^	0.000	0.712	(−0.000, 0.000)^1,2^	
		Time^3^	0.000	0.900	(−0.000, 3.218)^2^	

	*mean(Heat)*	Intercept	2.743	0.004	(1.738, 3.454)	186.497
		L vs. C	−0.115	0.312	(−0.418, 0.165)	
		H vs. C	0.066	0.988	(−0.275, 0.359)	
		Time (hr)	−0.034	0.008	(−0.006, −0.000)^2^	
		Time^2^	−0.003	0.216	(−0.000, 0.000)^1,2^	
		Time^3^	0.000	0.340	(−0.000, 0.000)^1,2^	

p* is the non-parametric *p*-value calculated as $P=2\;*\;min\left[P\left(\overset{ˆ}{\beta }<0|H_{0}\right),\;P\left(\overset{ˆ}{\beta }>0|H_{0}\right)\right]$, under the null hypothesis of β=0.

CI* is non-parametric 95% confidence interval obtained by the percentile bootstrap 95% confidence interval.

1 0.000 represents small positive values such as 0.000003 that were rounded up to 0.000 when only keeping 3 decimal digits.

2 −0.000 represents negative values such as −0.000003 that were rounded up to 0.000 when only keeping 3 decimal digits.

Time^2^ is squared time (mins).

Time^3^ is cubic time (mins).

C, control; L, Low dose; H, High dose; hr, hour; min, minutes

**Table 5.** Results for B-spline models of heat production (kcal/kg BW/hr) with linear, quadratic and cubicsplines.

Model	Outcome	Variable	$\overset{ˆ}{\beta }$	p*	CI*	AIC

(SMEM) (linear spline)	Heat	Intercept	2.754	0.560	(−0.292, 3.147)	3379.22
		L vs. C	−0.170	0.596	(−0.325, 0.295)	
		H vs. C	0.037	0.784	(−0.222, 0.309)	
		Time (mins)	0.000	0.648	(−0.001, 3.09)	

	*mean(Heat)*	Intercept	2.704	<0.0001	(2.604, 3.209)	162.114
		L vs. C	−0.159	0.268	(−0.359, 0.149)	
		H vs. C	0.082	0.744	(−0.246, 0.342)	
		Time (hr)	−0.016	0.004	(−0.001, −0.000)^2^	

(SMEM) (quadratric spline)	Heat	Intercept	3.064	0.568	(−0.272, 3.315)	3344.63
		L vs. C	−0.176	0.620	(−0.344, 0.263)	
		H vs. C	0.093	0.832	(−0.222, 0.319)	
		Time (mins)	−0.002	0.648	(−0.003, 0.000)^1^	
		Time^2^	0.000	<00001	(0.000, 3.297)^1^	

	*meag(Heat)*	Intercept	2.899	<0.001	(2.722, 3.364)	142.136
		L vs. C	−0.127	0.316	(−0.367, 0.179)	
		H vs. C	0.100	0.672	(−0.214, 0.36)	
		Time (hr)	−0.115	<0.0001	(−0.003, −0.001)	
		Time^2^	0.004	<0.0001	(0.000, 0.0000)^1^	

(SMEM) (cubic spline)	Heat	Intercept	2.923	0.552	(−0.276, 3.447)	3376.26
		L vs. C	−0.106	0.636	(−0.327, 0.321)	
		H vs. C	0.065	0.852	(−0.206, 0.36)	
		Time (mins)	−0.003	0.552	(−0.004, 0.000)	
		Time^2^	0.000	0.728	(−0.000, 0.000)^1,2^	
		Time^3^	0.000	0.640	(−0.000, 3.402)^2^	

	*meag(Heat)*	Intercept	2.909	<0.0001	(2.741, 3.427)	164.561
		L vs. C	−0.121	0.364	(−0.38, 0.174)	
		H vs. C	0.126	0.620	(−0.224, 0.368)	
		Time (hr)	−0.083	0.028	(−0.004, 0.000)	
		Time^2^	0.001	0.472	(−0.000, 0.000)^1,2^	
		Time^3^	0.001	1.000	(−0.000, 0.000)^1,2^	

p* is the non-parametric *p*-value calculated as $P=2\;*\;min\left[P\left(\overset{ˆ}{\beta }<0|H_{0}\right),\;P\left(\overset{ˆ}{\beta }>0|H_{0}\right)\right]$ under the null hypothesis of β=0.

CI* is non-parametric 95% confidence interval obtained by the percentile bootstrap 95% confidence interval.

1 0.000 represents small positive values such as 0.000003 that were rounded up to 0.000 when only keeping 3 decimal digits.

2 −0.000 represents negative values such as −0.000003 that were rounded up to 0.000 when only keeping 3 decimal digits.

Time^2^ is squared time (mins).

Time^3^ is cubic time (mins).

(C, control; L, Low dose; H, High dose; hr, hour; min, minutes)

## Data Availability

The datasets used and/or analyzed during the current study are available from the corresponding author on reasonable request.

## Abbreviations

AIC: Aikake information criteria
LMEM: linear mixed effects model
SMEM: semiparametric mixed effects model
TPBF: truncated power basis function
ZDF: Zucker diabetic fatty
